# *ANKLE1* as New Hotspot Mutation for Breast Cancer in Indian Population and Has a Role in DNA Damage and Repair in Mammalian Cells

**DOI:** 10.3389/fgene.2020.609758

**Published:** 2021-01-27

**Authors:** Divya Bakshi, Archana Katoch, Souneek Chakraborty, Ruchi Shah, Bhanu Sharma, Amrita Bhat, Sonali Verma, Gh. Rasool Bhat, Ashna Nagpal, Samantha Vaishnavi, Anindya Goswami, Rakesh Kumar

**Affiliations:** ^1^Shri Mata Vaishno Devi University, Katra, India; ^2^Cancer Pharmacology Division, Indian Institute of Integrative Medicine (CSIR) Jammu, Jammu, India; ^3^Academy of Scientific and Innovative Research (AcSIR), New Delhi, India; ^4^Department of Botany, Central University of Jammu, Jammu, India

**Keywords:** *ANKLE-1*, γH2AX, cancer, breast cancer, MCF-7 cell line, DNA damage

## Abstract

Breast cancer has replaced cervical cancer as being the most common and having the highest mortality among women in India. *ANKLE* gene is conserved among organisms during evolutionary succession and is a member of LEM family proteins in lower metazoans and is involved in critical functions in the nuclear architecture, gene expression and cell signaling. *ANKLE1* is the human orthologous of LEM-3 and is involved in DNA damage response and DNA repair. Whole Exome Sequencing (WES) of paired breast cancer samples was performed and *ANKLE1* was found to be a new possible hotspot for predisposition of breast cancer. The mass array genotyping for breast cancer variant rs2363956 further confirmed the *ANKLE1* association with the studied population of breast cancer. To elucidate the role of *ANKLE1* in DNA damage, it was knocked down in MCF-7 breast cancer cell line and the expression of γH2AX was assessed. *ANKLE1* knockdown cells displayed elevated levels of γ-H2AX foci in response to the cisplatin induced replication stress. The localization pattern of *ANKLE1* further emphasized the role of *ANKLE1* in DNA repair process. We observed that *ANKLE1* is required for maintaining genomic stability and plays a role in DNA damage and repair process. These findings provided a molecular basis for the suspected role of *ANKLE1* in human breast cancer and suggested an important role of this gene in controlling breast cancer development among women in India.

## Introduction

Cancer development is a multistage process involving several genetic alterations (Takashi et al., 1992). Plethora of carcinogens like ultraviolet radiations, ionizing radiations and toxic chemicals attack the DNA and create DNA lesions (Roos and Kaina, 2013). If unrepaired, the accumulation of these lesions might lead to precarious oncogenic progressions in the cell and enhance the cancer risk (Hoeijmakers, 2001). The development of cancers involves lacunae in the DNA repair pathways that are now being targeted for treatment processes (Gomes et al., 2017). Breast cancer is the most frequent type of cancer among women worldwide and is the leading cause of cancer related deaths (Miranda and Fidier, 2018). Breast cancer shares 14% of the cancer mortality burden worldwide and affects 1.3 million women worldwide (Lam et al., 2014). The most frequent mutations in breast cancer are associated with DNA repair mechanisms in the cell (Ciriello et al., 2013). Several genes *BRCA1*, *BRCA2, PALB2, CHEK2, PTEN, RAD51, RAD52, XRCC1, XRCC2* have been characterized for their involvement in hereditary or sporadic breast cancers (Walsh et al., 2006; Majidinia and Yousefi, 2017). In the recent years, the genome-wide association studies (GWAS) have helped in identification of many breast cancer susceptibility loci (Douglas et al., 2007). The breast cancer is found to be polygenic (Paul et al., 2002). The common alleles discovered by GWAS in the general population confer a higher risk in *BRCA1* and *BRCA2* mutations (Simon et al., 2007). Inherited mutations in these genes confer a lifetime risk of breast cancer (Paula et al., 2009). BRCA family of genes plays critical role in DNA Damage response and Repair.

SNP genotyping provides a powerful tool for identifying the contributors in complex disorders (Brookes, 1999). Mass array genotyping using MALDI-TOF spectrometry allows rapid genotyping of several hundred SNPs in the cost-effective manners (Oeth et al., 2009). Genotyping studies have highlighted several variations in the genes that might contribute in the breast cancer development. Exome sequencing has helped to decipher the genetic basis of several sporadic cancers and other human inherited diseases (Rabbani et al., 2012). In various cancer studies, the exome sequencing succeeded in identifying novel mutations of hereditary nature (Noetzli et al., 2015). Recent studies have indicated toward an increased predisposition to breast and ovarian cancer with small nucleotide polymorphisms in the human *ANKLE1* gene (Kristen et al., 2011; Lawrenson et al., 2016). The expression of *ANKLE1* in breast epithelial cells (Rosenbloom et al., 2012) and its presence in ovarian cancer indicated that *ANKLE1* might be regulating women cancers through hormonal mechanisms (Bolton et al., 2016). Whole exome sequencing has helped in identifying several loci within the *ANKLE1* gene, which account for autoimmunity disorders (Johar et al., 2015). The LEM family proteins are involved in nuclear architecture, gene expression, cell signaling etc. The mammalian genome study has spotted genes belonging to the LEM family termed as LEM3 and LEM4 also called the ankyrin repeat and LEM domain containing proteins 1 and 2, respectively (Brachner et al., 2011). *ANKLE1* or the *LEM3* protein is present in the hematopoietic tissue and cells. It is an evolutionarily conserved non membrane bound protein transported between cytoplasm and nucleus (Lacy et al., 2016). In *C. elegans*, the mutation in the *LEM-3* gene (mammalian orthologous of *ANKLE1* gene) has shown to develop extreme sensitivity to DNA damaging agents (Brachner and Foisner, 2014). The up-regulated expression of *ANKLE1* in DNA damage conditions confirms the role of *ANKLE1* under stress conditions. These studies suggest that *ANKLE1* might be a relevant factor in DNA damage response and DNA repair. Detailed studies on *ANKLE1* involvement in breast cancer for Indian population are unknown.

Here we report the detailed studies on WES and mass array genotypic of variant rs2363956 and observed that genetic variations in *ANKLE1* is associated with breast cancer population in India. Further, we provided evidence that *ANKLE1* is involved in DNA repair process in MCF-7 cell lines as the absence of *ANKLE1* resulted in an increased DNA damage. The cells exposed to DNA damaging agents showed *ANKLE1* localization in nucleus. These results together suggested that *ANKLE1* plays an important role in breast cancer development in Indian population most likely by regulation DNA repair in breast cancer cell lines.

## Methodology

### Study Materials

The recruitment of subjects of breast cancer cases was confirmed by histopathological examination, and those with any other type of cancer were excluded. A small number of samples were analyzed through WES. For SNP Mass Array Genotyping a total of 550 blood and tissue samples were recruited (150 cases and 400 controls). The details of the samples have been mentioned in Supplementary Table 1. The MCF-7 (Michigan Cancer Foundation-7) breast cancer cell line was used for the study. These cell lines were obtained from ATCC (American Type Culture Collection) United States. Gibco^TM^ RPMI 1640 (Roswell Park Memorial Institute) media was used for the MCF-7 cell culture. The media was supplemented with 10% FBS and 1% penicillin. The cells were grown at a temperature of 37°C in a 5% CO_2_ incubator up to 75–80% confluence. The study was approved by the Institutional Ethics Review Board (IERB) of SMVDU (SMVDU/IERB/18/70).

### Mutation Detection and Analysis

The samples were outsourced for WES at the Xcelris Labs Pvt. Limited, Ahmedabad, Gujarat, India. About 3 μg of the DNA isolated, using the Qaigen DNA isolation kit (Cat. No. 69504) was sent for whole exome sequencing. Illumina HiSeq 2000 with paired-end 100-bp reads was used for the Whole Exome Sequencing. For the tissue samples, DNA isolation was done using the Nectera rapid capture exome kit. 50 ng of DNA was then used for sequencing using Illumina HiSeq 2500 sequencer. Based on plethora of hotspots elucidated in WES and after validation through various bioinformatic tools, *ANKLE1* gene was chosen. The main criterion for the gene selection was its consistent presence in Breast Cancer samples.

Similarly, the mass array for SNP genotyping was carried out for rs2363956 variant within the *ANKLE1* gene by using Sequenom Mass Array iPLEX. The 550 samples were employed (150 breast cancer cases and 400 control samples) and studied for the genotypes at the location rs2363956 on the *ANKLE1* gene. Genotypes were analyzed based on the ratio of MALDI-TOF spectrometer. SNP genotyping and SNP allele frequency were calculated using software.

### Cell Lysate Preparation and Western Blotting

The cells were given 25 μM cisplatin treatment and rested for time intervals of 0, 4, 8, 16 and 24 h. After the respective treatments, the cells were centrifuged and mixed with a lysis buffer to obtain the cell lysate. The total protein concertation was determined using Lowry method. The equal amount of total proteins was separated on SDS-PAGE, immunoblotted using antibodies against *ANKLE1* (Merck Inc.) (Cat. No. HPA073498-100UL), γ-H2AX and β-Actin using methods as described (Merck, 2020). In brief, proteins separated on SDS-PAGE were transferred to nitrocellulose membrane and blocked with 5% BSA. Thereafter adding primary antibodies in the dilution of 1:1,000, the blots were washed and probed with the respective secondary antibodies (Merck Inc.) coupled with horseradish peroxidase (Merck Inc.). The secondary antibody in a dilution of 1:2,000 was used. The bands were developed using the chemiluminescence reagent (Millipore) and captured onto Biomax light film (Eastman Kodak Co., Rochester, NY).

### Immunocytochemistry (ICC)

The cells were subjected to ICC to assess the localization and expression of *ANKLE1* and γH2AX. The cells cultured in chambered slides were fixed using paraformaldehyde. After washing the cells with PBS, permeabilization was done using 0.3% of Triton-X for 10 min. The cells were then subjected to primary anti-Rabbit and anti- Mouse antibody (*ANKLE1*, Merck Inc.) treatment overnight for 3 h, at 4°C, washed and then incubated with secondary antibody for *ANKLE1* and γH2AX for 2 h. at RT. DAPI was used as a counter stain. After pouring the mounting media and coverslip placement the visualization was done with 2X Floid^®^ microscope (FLoid Cell Imaging Station).

### *ANKLE1* siRNA Knockdown

MCF-7 cells were seeded in six well chamber slides and transfected using X-treme Gene^TM^ (Merck Inc.). *ANKLE1* siRNA (Merck Inc.) was employed to knockdown the gene expression. RNA scramble was used as siRNA control. These cells were checked for expression of *ANKLE1* immunoblotting. Following the siRNA treatment, the cells were treated with cisplatin and the cell lysates were obtained and used for immunoblotting and immunocytochemistry. Supplementary Table 3 shows the siRNA sequences used.

### Statistical Analysis

The values were subjected to densitometry analysis and *t*-test to analyze their statistical significance. All images were representative of three fields. The relative increase in the protein expression of *ANKLE1* at 4–24 h duration, for the western blot, were checked for statistical significance and found to be statistically significant with *p* < 0.0001. The relative decrease in the γH2AX expression with the increase in the *ANKLE1* expression was found to be statistically significant (*p* < 0.0001).

### Ethical Clearance

The study was approved by the Institutional Ethics Review Board (IERB) of Shri Mata Vaishno Devi University (SMVDU) (SMVDU/IERB/18/70). Written informed consent was obtained from each participant before conducting the study. All experimental protocols were conducted according to the guidelines and regulations set by IERB, SMVDU.

## Results

### WES Analysis Showed Mutant in *ANKLE1*

Plenitude of variations outside of the limited known pool are involved to impede the DNA repair processes. To better understand these variants that might be directly or indirectly involved in the DNA damage process, thus allying cancer progression, we performed the whole exome sequencing of the breast cancer samples. The effect of the variation was predicted based on their score levels. We assessed several gene variants and observed certain variants to be consistent in several samples. Located on chromosome 19, *ANKLE1* showed varied exonic, intronic, intergenic and upstream variations (Supplementary Table 2).

### Mass Array SNP Genotyping Showed Higher Frequency of GT Over GG in *rs2363956*

We next sought to determine the other potential repetitive variant locus of *ANKLE1* gene, rs2363956. Another locus on the *ANKLE1* elucidated in the whole exome sequencing was studied through the SNP Mass array genotyping. The studied variant rs2363956, on the *ANKLE1* gene, is a missense type of variant with T > G change. On comparing the difference of expression between genotypes GT and GG at rs2363956 in *ANKLE1* gene through the mass array SNP genotyping of subjects, we found that the genotype GT confers protection to the individual as compared to the genotype GG. It was found that the allele T within the rs2363956, with an OR of 0.7011 indicated a low risk of breast cancer. The allele frequency of allele T in cases was 0.37 and 0.45 in controls. The allele frequency of allele G was 0.63 in cases and 0.55 in controls. Thus, the genotype GT confers protection to the individual as compared to the GG genotype. The genotypes followed the Hardy Weinberg equilibrium (H.W.E, *p*-value 0.732) and were statistically significant (*p* = 0.02246) to our population. Data suggests that breast cancer is more frequent in patients carrying rs2363956 GT genotype than those carrying GG genotype.

### DNA Damage Responsive Increase in ANKLE1 Protein in MCF-7 Cells

**FIGURE 1:**
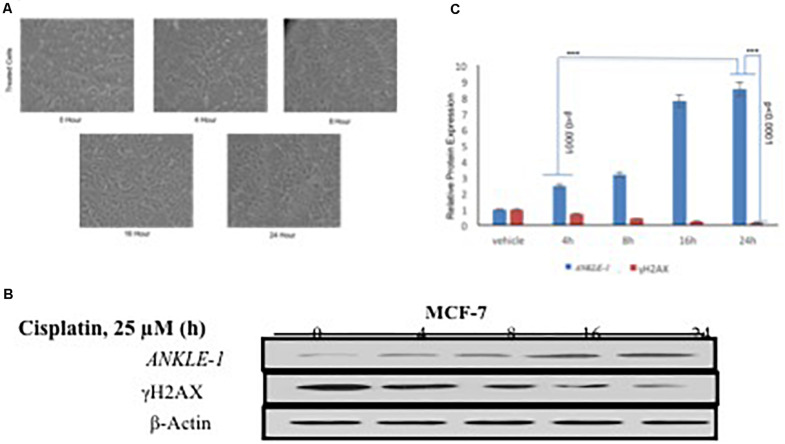
**(A)** MCF-7 Breast Cancer cell line at different time interval of cisplatin treatment. **(B)** Western blotting for *ANKLE1*, γH2AX and β-Actin. **(C)** Expression levels of *ANKLE1* & γH2AX at different time intervals.

We further explored the functional significance of *ANKLE1* gene after treatment with cisplatin, a known DNA damaging drug. MCF-7 cell line was treated with 25 μM cisplatin for different time intervals: 0, 4, 8, 16, and 24 h. Along with the increase in the treatment time of cisplatin, a slight increase in the cell volume, that is indicative of DNA damage, was seen. After encountering the stress stimulus triggered by cisplatin, the cells increase in volume and swell (Fulda et al., 2010). Cisplatin acts as a DNA damaging agent thus a potent stress signal for the cells. Figure 1A shows the images of the cells captured at different time interval of treatment. Cells treated at 0 h serve as control, whereas the cells captured at 24 h treatment seem to be inflated.

To investigate the contribution of *ANKLE1* gene in DNA repair in relation to breast cancer we assessed the expression levels under different exposure time intervals. Treated cells were analyzed for protein expression through western blotting. It was found that with the increase in the time of cisplatin treatment the expression of *ANKLE1* protein subsequently increased in the cells. The expression of *ANKLE1* was found to be gradually increasing with the increase in the treatment time, highest being at 24 h. However, the expression of γH2AX was inversely related to the *ANKLE1* expression levels. A high γH2AX was found where the *ANKLE1* expression was low and vice versa. The increased expression of γH2AX signals for DNA damage, however, with the increase in the *ANKLE1* expression the γH2AX was found to be relative decreased (Figure 1B). β-Actin served as a control and had a leveled expression. The values were subjected to *t*-test to analyze their statistical significance. The relative decrease in the γH2AX expression with the increase in the *ANKLE1* expression was studied. Figure 1C represents the statistical values of *ANKLE1* and γH2AX expression. The values were found to be statistically significant (*p* < 0.0001).

### DNA Damage Changes ANKLE1 Protein Localization

Given the key role of *ANKLE1* in breast cancer, we speculated that *ANKLE1* might be localized near the nuclear membrane considering its shuttling property inside the cytoplasm and nucleus. To analyze the localization and expression of the proteins under study, immunocytochemistry was performed. Vehicle served as the control, i.e., cells without the cisplatin treatment. Anti-Rabbit secondary antibody was used for *ANKLE1* and florescence was observed at 488λ (green color). Anti-Mouse secondary antibody was used for γH2AX and florescence was observed at 488λ. DAPI served as the counter stain and stained the nucleus (blue color).

**FIGURE 2:**
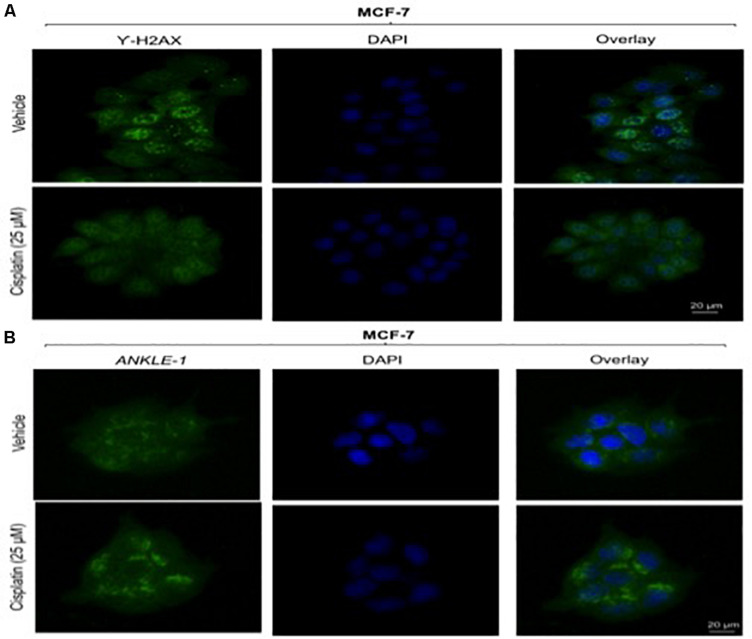
**(A)** Image showing ICC images of γH2AX in vehicle and treated cells. **(B)** Image showing ICC images of *ANKLE1* in vehicle and treated cells. ICC was performed to check the expression and localization of the proteins. The cells were fixed using 4% paraformaldehyde for 15 min at RT. Permeabilization was done using 0.3% Triton X for 10 min at RT and Blocking was done using PBS for 30 min. After addition of primary antibody (1:100) and secondary antibody (1:500) visualization was done in FLoid^®^ Cell Imaging Station.

As shown in Figure 2B, the expression of *ANKLE1* increased substantially, when the cells were treated with 25 μm of cisplatin, as compared to the vehicle. The overlay images showed the expression of *ANKLE1* inside the nucleus and in the cytoplasm, being highest around the nuclear membrane. Figure 2A shows that the expression of γH2AX was mitigated in cells that were treated with 25 μm cisplatin (with a higher expression of *ANKLE1*) as compared to the untreated cells. DAPI served as the counter stain and stained the nucleus. The overlay images showed the expression of γH2AX in the nucleus. The decrease in the expression of γH2AX corresponded to the increase in the *ANKLE1* expression.

### Knockdown of *ANKLE1* Gene Using si*ANKLE1*

**FIGURE 3:**
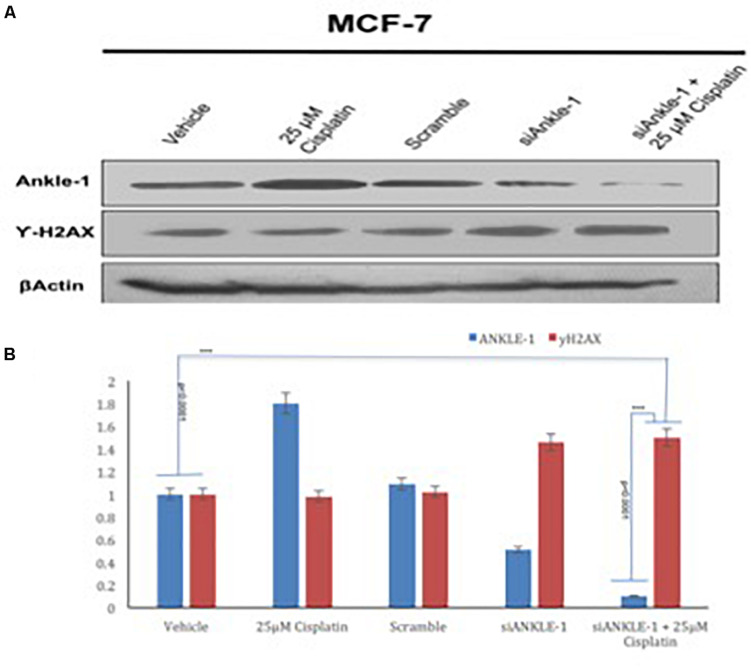
**(A)** Western blot for *ANKLE1*, γH2AX and β-Actin. **(B)** Expression levels of *ANKLE1* & γH2AX subsequent to treatments.

The *ANKLE1* gene was knocked down using si*ANKLE1* and the expression of the proteins was studied. We found that as compared to the vehicle or the control, the expression of *ANKLE1* was increased when the cells were treated with 25 μm cisplatin (Figure 3A). The expression of the scramble was equivalent to the expression of the control. The si*ANKLE1* treated cells showed a decreased level expression of *ANKLE1* as compared to the vehicle/control. The *ANKLE1* expression failed to increase when the si*ANKLE1* treated cells were treated with 25 μm cisplatin. The expression of γH2AX was decreased when the cells were treated with 25 μm cisplatin. However, when the cells were treated with si*ANKLE1* the expression of the γH2AX increased and elevated further when the si*ANKLE1* treated cells were treated with 25 μm cisplatin, nuancing toward an enhanced DNA damage in absence of *ANKLE1* protein.

Adequate levels of β-Actin were observed in the cells and served as a positive control. As shown in Figure 3B, the values were found to be statistically significant (*p* < 0.0001).

### Immunocytochemistry (ICC) Analysis After Treatment With si*ANKLE1*

**FIGURE 4:**
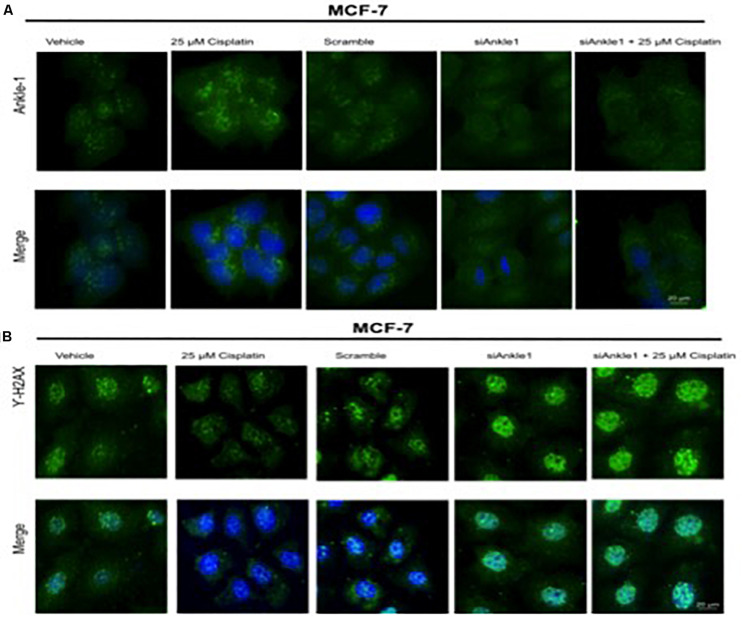
**(A)** Image showing ICC images of *ANKLE1* in vehicle and treated cells. **(B)** Image showing ICC images of γH2AX in vehicle and treated cells. The cells were treated with siANKLE1 and their expression was analyzed. The treated cells were fixed on the chamber slides by using 4% paraformaldehyde for 15 min at RT. The cells were permeabilized using 0.3 Triton X for 10 min at RT and Blocking was done using PBS for 30 min. After addition of primary antibody (1:100) and secondary antibody (1:500) visualization was done in FLoid^®^ Cell Imaging Station.

To further test whether the ability of the expression of the protein was affected we performed immunocytochemistry. The knockdown of the target gene was performed using si*ANKLE1* and immunocytochemistry study was done. Figure 3B shows the *ANKLE1* protein’s expression in the treated and untreated cells, in the absence and presence of *siANKLE1.* As compared to the vehicle/control the treated cells showed an increased expression of *ANKLE1* protein. The scramble had a comparable expression to the control. When the cells were treated with si*ANKLE1*, the expression of *ANKLE1* lowered and further decreased when si*ANKLE1* cells were treated with 25 μm cisplatin (Figure 4B).

Also, the expression of γH2AX was analyzed when the cells were treated with si*ANKLE1* (Figure 4A). γH2AX expression, as verified previously, decreased when the cells were treated with 25 μm cisplatin. However, its expression was elevated when the cells were treated with si*ANKLE1* and was increased further when the si*ANKLE1* cells were treated with 25 μm cisplatin showing a higher DNA damage in the cells. The merged images show the presence of γH2AX in nucleus by staining with DAPI, which stains the nucleus. ICC images correlated with the expression levels of si*ANKLE1* and γH2AX and highlighted the si*ANKLE1* location inside the nucleus and around the nuclear membrane.

## Discussion

The aim of the study was to evaluate the role of *ANKLE1*, identified through whole exome sequencing, in DNA repair process. We have reported the role of *ANKLE1* in DNA repair process, it’s mitigating action on the DNA damage in MCF-7 cell lines and an elevated DNA damage with knockdown of *ANKLE1* gene. In the present study we also studied the polymorphic variant of *ANKLE1* or the LEM3 gene. Whole-exome sequencing is a diagnostic approach for the identification of molecular defects in patients with suspected genetic disorders (Yang et al., 2013). *ANKLE1* gene is the human ortholog of LEM-3 gene and is conserved among species. *ANKLE1* contains a GIY-YIG endonuclease domain that is also present in the endonuclease SLX-1. *ANKLE1* like SLX-1 is involved in resolving the holliday-junction (Wyatt and West, 2014) through it’s resolvase activity and takes part in the Homologous recombination process of DNA repair (Saito et al., 2013). Several studies have implicated the mutations in *ANKLE1* gene to the development of breast and ovarian cancers indication its role in the DNA damage repair process (Bolton et al., 2010; Stevens et al., 2011). In this study we have demonstrated that *ANKLE1*, a potential DNA repair candidate, repairs the DNA damage caused by the treatment of MCF-7 cells with cisplatin. The role of H2AX is conspicuous with it being a biomarker of the DNA damage in the cells (Linda and Yang, 2008). The double strand break in the DNA leads to the phosphorylation of H2AX into γH2AX triggering it as being a initiator DNA damage repair machinery (Kobayashi, 2004). The phosphorylation of serine 139 on histone H2AX (Emmy et al., 1997) is rapid process, propagating swiftly from double stranded breaks (Lowndes and Toh, 2005). Interestingly, we found that when the cells were treated with cisplatin, a DNA damaging agent, the γH2AX subsequently decreased in the cells. This decrease in the γH2AX was relatable to the corresponding increase in the *ANKLE1* expression in the cells. *ANKLE1* is involved in the DNA damage response and DNA repair pathways (Brachner and Foisner, 2014). Our studies showed that when expression of *ANKLE1* was knocked down, the expression of γH2AX got elevated, indicating a higher DNA damage in absence of *ANKLE1* protein. Although it would be over ambitious to imply that *ANKLE1* is directly involved in DNA repair process, however, we suggest a collaborative effort of DNA repair proteins, working in conjugation to achieve the DNA repair target. *ANKLE1* has been shown to bind with the BAF family of proteins, which are shown to be involved in gene regulation processes (Brachner et al., 2012). The induction of DNA damage by cisplatin potentially triggered the *ANKLE1* for the DNA damage repair and lowered the expression of γH2AX. Since γH2AX gives the measure of DNA damage in the cell, we can state the role of *ANKLE1* in DNA repair process. The *ANKLE1* gene offers a magnanimous prospect in the cancer therapeutics. The overexpression of *ANKLE1* in mammalian cells has shown to trigger the DNA damage response, whereas, knocking out the LEM-3 gene which is ortholog of *ANKLE1*, in *C. elegans* attributes to an increased sensitivity to DNA damaging agents (Brachner and Foisner, 2014). Thus *ANKLE1* poses to be a promising candidate to explore for curtailing the extensive spread of the neoplastic disease.

## Conclusion

Breast cancer is a multifactorial disease that has become the most common cancer in Indian women. Despite its common prevalence there is a lacuna in the data regarding the genetic framework of the disease especially in the population of Jammu and Kashmir. The Whole Exome Sequence study gave us an insight into thousands of single nucleotide variations in the genome of breast cancer patients. Few variants were found to be consistent in numerous samples under study. Through the mass array genotyping we confirmed the disease-associated genotype in 550 samples The knockdown study performed with siRNA *ANKLE1*, highlighted the increase in *ANKLE1* expression with the DNA damaging agent cisplatin, however, with the si*ANKLE1* (siRNA *ANKLE1*) treatment, the *ANKLE1* expression decreased. γH2AX was conversely related to the *ANKLE1* expression degree. The siRNA *ANKLE1* treated cells showed a basal expression of *ANKLE1*, which failed to increase with the cisplatin treatment. On the contrary, when treated with siRNA *ANKLE1*, the γH2AX expression was found to be elevated in the cells indicating a high DNA damage. The localization of the *ANKLE1* protein was found to be concentrated near the nuclear membrane congruent with its property of shuffling between nucleus and cytoplasm. Since only few proteins associated with DNA organization or repair have the NIS (Nucleus Import signal) enter the nucleus, this property further establishes the role of *ANKLE1* in DNA damage response. Since *ANKLE1* has been a player before in some cancers, further intensive study could be done to investigate the role of *ANKLE1* and its potential role in DNA repair mechanism in various cancers. *ANKLE1* gene be further validated and exploited for developing a novel approach toward breast cancer diagnosis and treatment. Further the limitations of this study should be catered to by studying the expression of *ANKLE1* in triple negative cells along with the checking the co-occurrence of other markers like ATM, P53, NF-κB, and TWIST.

## Data Availability Statement

The original contributions presented in the study are included in the article/Supplementary Material, further inquiries can be directed to the corresponding author/s.

## Ethics Statement

The studies involving human participants were reviewed and approved by the Institutional Ethical Review Board (IERB) (SMVDU/IERB/18/70) of Shri Mata Vaishno Devi University. The patients/participants provided their written informed consent to participate in this study. Written informed consent was obtained from the individual(s) for the publication of any potentially identifiable images or data included in this article.

## Author Contributions

RK designed the study. DB and AK performed the experiments and extrapolated results. DB wrote the manuscript. SC helped with the experimental work. AN, RS, SV, AB, GB, and BS helped in sample collection. AG and SV helped in guiding the study. All authors contributed to the article and approved the submitted version.

## Conflict of Interest

The authors declare that the research was conducted in the absence of any commercial or financial relationships that could be construed as a potential conflict of interest.
