# Supplementary material for: ANKLE1 as New Hotspot Mutation for Breast Cancer in Indian Population and Has a Role in DNA Damage and Repair in Mammalian Cells
Source: Front Genet. 2021 Jan 27;11:609758. doi: 10.3389/fgene.2020.609758 (PMC7873468; doi:10.3389/fgene.2020.609758)
Supplement: Supplementary file 1 [file Data_Sheet_1.docx]

**ANKLE-1 as new hotspot mutation for Breast cancer in Indian population and has a role in DNA Damage and repair in mammalian cells.**

**Authors**: Divya Bakshi^1^, Archana Katoch^3^, Souneek Chakraborty^3^, Ruchi Shah^1^, Bhanu Sharma^1^, Amrita Bhat^1^, Sonali Verma^1^, Gh. Rasool Bhat^1^, Ashna Nagpal^1^, Samantha Vaishnavi^3^, Anindya Goswami^2^, **Rakesh Kumar**^*1^.

^1^Shri Mata Vaishno Devi University, Katra.  ^2^Department of Plant Sciences, Central University of Jammu. India. ^3^Indian Institute of Integrative Medicine, Jammu. India

^*^**Corresponding Author**. Email: kumar.rakesh@smvdu.ac.in

**Supplementary Section**

**TABLE 1**: Clinical details and allele frequency of cases and controls.

| **CHARACTERSTICS** | **CASES** | **CONTROL** | **p VALUE** |
| --- | --- | --- | --- |
| **Age [±SD]**  **in years** | 50.4 ± 11.6 | 55.1 ± 14.5 | 0.0004 |
| **BMI [±SD]**  **in Kg/m^2^** | 22.1 ± 2.32 | 25.20 ± 4.2 | 0.002 |
| **Postmenopausal** | 56% | 68% | |
| **Premenopausal** | 44% | 32% | |

**TABLE 2**: Exonic and Intronic ***ANKLE-1*** SNP’s in all WES samples.

| **Sample No.** | **SNP** | **ExonicFunc.refGene** | **Reference Allele** | **Altered Allele** | **Function** |
| --- | --- | --- | --- | --- | --- |
| I | rs8100241 | nonsynonymous SNV | G | A | exonic |
|  | rs8108174 | nonsynonymous SNV | T | A | exonic |
|  | rs2363956 | nonsynonymous SNV | T | G | exonic |
|  | rs891017 | nonsynonymous SNV | A | C | exonic |
|  | rs11086065 | nonsynonymous SNV | A | G | exonic |
|  | rs11882562 | synonymous SNV | C | G | exonic |
|  | NA | synonymous SNV | C | A | exonic |
|  | rs751599 | synonymous SNV | T | C | exonic |
|  |  |  |  |  |  |
| II | rs11882562 | synonymous SNV | C | G | exonic |
|  | rs751599 | synonymous SNV | T | C | exonic |
|  | rs891017 | nonsynonymous SNV | A | C | exonic |
|  | rs11086065 | nonsynonymous SNV | A | G | exonic |
|  | rs10425939 | nonsynonymous SNV | C | T | exonic |
|  | rs66753001 | NA | A | G | intronic |
|  |  |  |  |  |  |
| III | rs66753001 | NA | A | G | intronic |
|  | rs891017 | nonsynonymous SNV | A | C | exonic |
|  | rs11086065 | nonsynonymous SNV | A | G | exonic |
|  | rs10425939 | nonsynonymous SNV | C | T | exonic |
|  | rs11882562 | synonymous SNV | C | G | exonic |
|  | rs751599 | synonymous SNV | T | C | exonic |
|  |  |  |  |  |  |
| IV | rs891017 | nonsynonymous SNV | A | C | exonic |
|  | rs11086065 | nonsynonymous SNV | A | G | exonic |
|  | rs10425939 | nonsynonymous SNV | C | T | exonic |
|  | rs11882562 | synonymous SNV | C | G | exonic |
|  |  |  |  |  |  |
| V | rs35586766 | nonsynonymous SNV | G | A | exonic |
|  | rs8100241 | nonsynonymous SNV | G | A | exonic |
|  | rs8108174 | nonsynonymous SNV | T | A | exonic |
|  | rs2363956 | nonsynonymous SNV | T | G | exonic |
|  | rs891017 | nonsynonymous SNV | A | C | exonic |
|  | rs66753001 | NA | A | G | intronic |
|  | rs11086065 | nonsynonymous SNV | A | G | exonic |
|  | rs11882562 | synonymous SNV | C | G | exonic |
|  | rs751599 | synonymous SNV | T | C | exonic |
|  | rs10425939 | nonsynonymous SNV | C | T | exonic |
|  |  |  |  |  |  |
| VI | NIL |  |  |  |  |
|  |  |  |  |  |  |
| VII | rs11882562 | synonymous SNV | C | G | intronic |
|  | rs751599 | synonymous SNV | T | C | exonic |
|  | rs891017 | nonsynonymous SNV | A | C | exonic |
|  | rs11086065 | nonsynonymous SNV | A | G | exonic |
|  | rs10425939 | nonsynonymous SNV | C | T | exonic |
|  | rs66753001 | NA | A | G | exonic |
|  |  |  |  |  |  |
| VIII | rs751599 | synonymous SNV | T | C | exonic |
|  | rs891017 | nonsynonymous SNV | A | C | exonic |
|  | rs11086065 | nonsynonymous SNV | A | G | exonic |
|  | rs10425939 | nonsynonymous SNV | C | T | exonic |
|  | NA | nonsynonymous SNV | C | T | exonic |
|  | rs66753001 | NA | A | G | intronic |

**Table 3:** siRNA gene pool duplex

| **Oligonucleotide Name** | **Tm°** | **GC%** | **Sequence (5’-3’)** |
| --- | --- | --- | --- |
| si*ANKLE-1* S1 | 60.8 | 47.6 | CGUGUAUUGUGGAAGCCCU |
| si*ANKLE-1* A1 | 62.3 |  | AGGGCUUCCACAAUACACG |
| si*ANKLE-1* S2 | 55.6 | 38.0 | CUAAUUUGGUGCUAGAGGA |
| si*ANKLE-1* A2 | 57.1 |  | UCCUCUAGCACCAAAUUAG |
| si*ANKLE-1* S3 | 57.1 | 42.8 | CUGUGUCUGACUUGGAGUU |
| si*ANKLE-1* A3 | 58.6 |  | AACUCCAAGUCAGACACAG |
| si*ANKLE-1* S4 | 64.0 | 52.3 | CUCUACAGGUGGCAGGGAA |
| si*ANKLE-1* A4 | 65.1 |  | UUCCCUGCCACCUGUAGAG |
